# Digital transfer in radiation oncology education for medical students—single-center data and systemic review of the literature

**DOI:** 10.1007/s00066-022-01939-w

**Published:** 2022-04-29

**Authors:** Michael Oertel, Niklas Benedikt Pepper, Martina Schmitz, Jan Carl Becker, Hans Theodor Eich

**Affiliations:** 1 Department of Radiation Oncology, University Hospital Muenster, Albert-Schweitzer-Campus 1, building A1, 48149 Muenster, Germany; 2 Institute of Anatomy and Vascular Biology, University of Muenster, Vesaliusweg 2–4, 48149 Muenster, Germany; 3 Department of Medical Education (IfAS), University of Muenster, Albert-Schweitzer-Campus 1, building A6, 48149 Muenster, Germany

**Keywords:** Medical education, e-learning, National Competence Based Learning Objectives Catalogue for Medicine (NKLM), Flipped classroom, Masterplan Medizinstudium

## Abstract

**Purpose:**

Modern medical education demands innovative, competence-orientated concepts. The forced digital transfer of teaching due to the coronavirus pandemic also affected radiation oncology (RO). The following analysis investigates whether the attractivity of RO teaching at our faculty could be maintained during the pandemic and which possibilities exist to involve students (in active learning). The latter aspect is further elaborated on a broader scale by a systemic review of the literature on competence-orientated digital education.

**Methods:**

Evaluation results and participation rates of clinical lectures in radiation oncology (RO) were analyzed between the winter semester 2018/2019 and the summer semester 2021. A systemic review of the literature on digital education in RO for medical students was conducted.

**Results:**

Concerning evaluation results, a significant improvement for the 7th and 9th semesters was observed in comparison between the pre-pandemic and pandemic semesters (*p* = 0.046 and *p* = 0.05, respectively). Overall participation rates did not differ. However, the number of students attending > 75% of classes in the respective semester increased significantly between the pre-pandemic and pandemic period (median values: 38 vs. 79%, *p* = 0.046; 44 vs. 73%, *p* = 0.05; 45 vs. 64%, *p* = 0.05; 41 vs. 77%, *p* = 0.05; 41 vs. 71%, *p* = 0.05, for the 6th to 10th semester, respectively).

**Conclusion:**

The analysis demonstrates the possibility of efficient digital transfer of a core curriculum in RO to the digital era, with a more continuous participation of students. This transfer may enable amelioration of teaching quality and the introduction of innovative and interactive concepts in accordance with the literature.

## Introduction

Modern education is pivotal for the formation of competent young medical professionals [1]. In recent years, there has been a considerable shift from simple knowledge acquisition to competence-based learning [2, 3]. This trend is supported by legislation: in Germany, the government has introduced the *Masterplan Medizinstudium 2020*, which focusses on practical education as well as longitudinal and interdisciplinary concepts [4]. Radiation oncology (RO) has a cardinal role in the treatment of cancer patients, being indicated in around half of all oncological patients during the course of disease [5]. The current pandemic caused by the severe acute respiratory syndrome coronavirus 2, a member of the RNA-containing *Coronaviridae* family, has struck global health systems with the consecutive disease (COVID-19), causing 456.8 million cases and resulting in 6.0 million deaths worldwide till the submission of this work [6, 7]. Despite this evolution, RO treatment is highly prioritized, emphasizing the subject’s importance for modern oncological concepts [8–10]. Concerning teaching, federal laws in Germany enabled universities to remain open and maintain teaching activities during the pandemic in 2020 [11]. However, with vaccines being unavailable, our faculty, among others, decided to abandon traditional teaching “in presence” and replace it with a digital concept. This forced digital transfer was in accordance with recommendations from other institutions [12] and had to be performed in just a few weeks. Digital concepts (e-learning) may entail didactic ameliorations by introducing additional (multimedia) material to enhance the learning experience but also by standardization of content and delivery [13]. Nevertheless, the impact of e‑learning on participation rates and evaluation results in medical education is yet to be defined. One apprehension was that the remote character of digital education would result in a deterioration of evaluation results and participation rates. Consequently, the current evaluation analyzes the effect of digital transformation on the RO curriculum at our faculty. It further investigates additional digital concepts worldwide and discuss their implications, focusing on active learning. The systemic review aims at identifying the available evidence on the subject but also at highlighting innovative teaching projects. To our knowledge, this is the first systemic review of the existing literature on digital education for medical students in RO.

## Materials and methods

### Teaching

The teaching concept of RO at our institution constitutes a longitudinal curriculum starting from the third semester, which is dedicated to basic subjects like anatomy and biochemistry. In an interdisciplinary training project (“Anatomy and Imaging” [14]) students are introduced to RO via interactive scenarios fostering transfer of anatomical knowledge to clinical cases. As a teaching subject, RO is summarized in the cross-sectional subject “imaging procedures, radiation therapy, and radiation protection” together with “(diagnostic) radiology and nuclear medicine.” The fifth semester (first clinical semester) provides fundamental knowledge of RO concepts, radiation biology, and physics, and acts as a “basic” semester for the following clinical lectures (6th–10th semester) dedicated to distinct entities (lung, hematology, gastroenterology, head and neck cancer, sarcoma, gynecology, urology, pediatric cancers). The attendance of lectures at our faculty is optional, in contrast to compulsory seminars and practical training sessions, which take place in the fifth semester only and are not analyzed in this work.

Beginning from the summer semester 2020, a lockdown was applied to all universities in our state, with teaching limiting to remote online courses. Within a few weeks, all lectures, seminars, and practical training sessions had to be transformed digitally. Conferences were held via Zoom (Zoom Video Communications, Inc., San José, CA, USA) on a university server to enable patient data transfer, if necessary. Since the winter semester 2021/2022, a hybrid concept has been implemented: students can attend seminars and lectures in presence if vaccinated, tested, or recovered. Furthermore, the session is transmitted digitally to attendees at home.

### Evaluation and statistical evaluation

Evaluation of each course at our faculty is compulsory after completion of the respective semester and performed digitally via an online form (EVALuna Münster, version 3.0, Binary Design GmbH, Münster, Germany) on a 101-point Likert scale (0 being the best, 100 being the worst grade). From the beginning of the summer semester 2020, lectures were digitalized, and digital transfer was additionally rated on an 11-item Likert scale (from −5 to +5, with −5 being the best grade). Evaluation results and participant numbers between the winter semester 2018/2019 and the summer semester 2021 were assessed, the first three semesters being classified as “pre-pandemic” and the following semesters as “pandemic.” Our analysis covers results from the course “Anatomy and Imaging” and lectures of the 6th–10th semester. The 5th semester, a basic semester, was intentionally left out as it displays some overlap to related disciplines (radiology, nuclear medicine) which are frequently mistaken for one another in the evaluation.

Statistical analysis was done using the program SPSS (IBM, Armonk, NY, USA) version 28. Mann–Whitney *U*-tests were calculated to test for differences between the pre-pandemic and pandemic semesters regarding evaluation results, and participation numbers with a *p*-value ≤ 0.05 considered as significant. The exact *p*-value was used for participation numbers. Due to similar values (ties) in the analysis for participation rate > 75% and most evaluation results, the asymptotic *p*-value was used for these categories.

### Systemic review

**Fig. 1:**
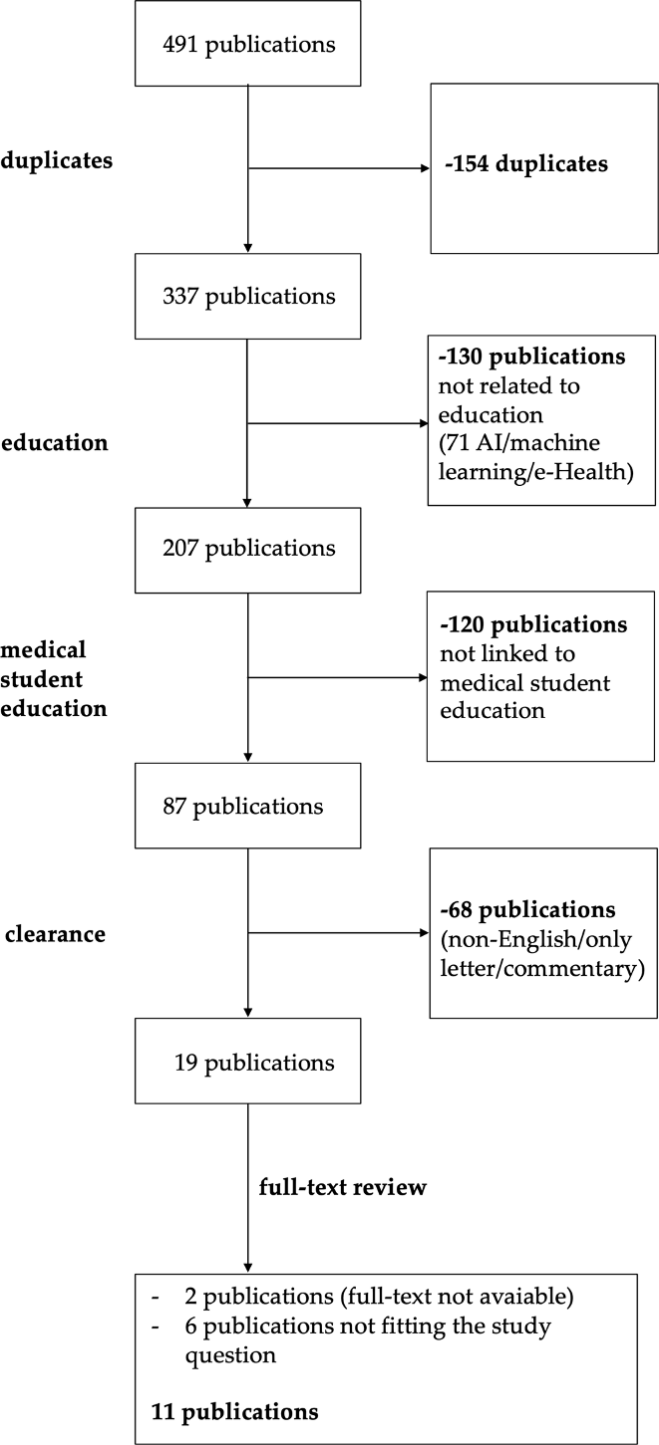
**Search strategy**. Search and exclusion strategy for the systemic review of digital education for medical students in radiation oncology. *AI* artificial intelligence

The objective of the systemic review was to assess feasibility and efficacy of digital education for medical students in RO. To identify relevant publications, a systemic review according to the PRISMA criteria has been carried out [15]. We searched the databases “PubMed” and “Scopus” on November 6 (PubMed) and November 9 (Scopus) 2021. For PubMed, the search was done with the following term(s): (″radiation oncology″[Title/Abstract] OR ″radiation therapy″[Title/Abstract] OR radiotherapy[Title/Abstract] OR ″therapeutic radiology″[Title/Abstract] OR ″therapeutic radiography″[Title/Abstract] OR ″therapy radiology″[Title/Abstract] OR ″therapy radiography″[Title/Abstract] OR ″radiotherapy″ [Subheading]) AND (((education[Title/Abstract] OR teaching[Title/Abstract] OR training[Title/Abstract] OR learning[Title/Abstract]) AND (digital[Title/Abstract] OR virtual[Title/Abstract] OR ″Digital Technology″[Mesh])) OR ″Technology/education″[Mesh]), whereas Scopus was searched via ″TITLE-ABS (″radiation oncology″ OR ″radiation therapy″ OR radiotherapy OR ″therapeutic radiology″ OR ″therapeutic radiography″ or ″therapy radiology″ or ″therapy radiography″) AND TITLE-ABS (learning OR education OR teaching OR training) AND TITLE-ABS (virtual OR digital)″. All searches were performed by the first author and repeated by the second author to confirm results. Articles were first scanned via title and abstract, excluding all non-English papers, duplicates, and publications only available as conference papers. Relevant publications were then read in detail by the first author and discarded or included, as appropriate. Educational papers focusing on groups other than medical students (e.g., nurses or radiation therapists) are not included in the final presentation, nor are publications on post-graduate education. The search and exclusion strategy is illustrated in Fig. 1.

## Results

**Fig. 2:**
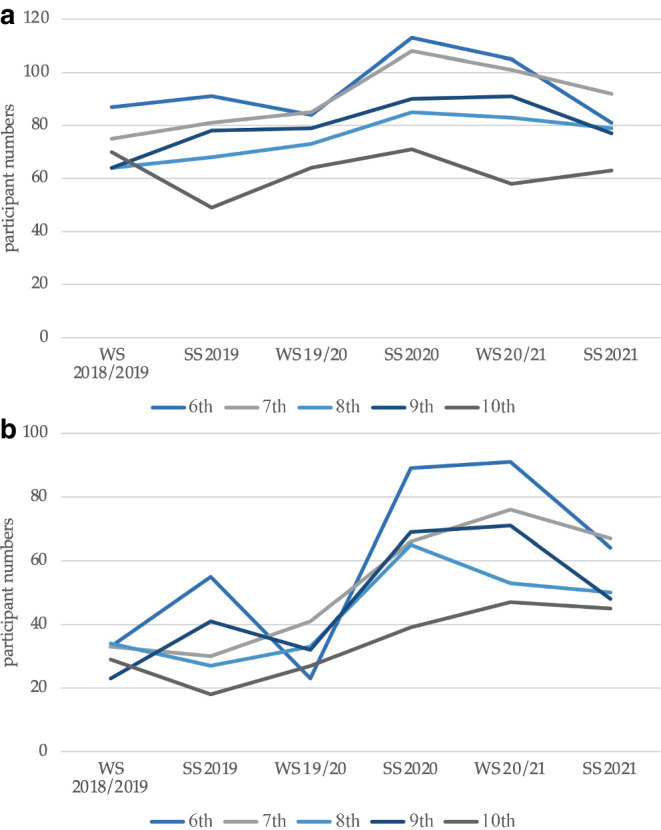
**Development of participant numbers.** Number of participating students in the 6th to 10th from winter semester 2018/2019 to summer semester 2021. **a** Total participant numbers, **b** number of participants attending > 75% of the respective courses in the semester. *SS* summer semester, *WS* winter semester

During the study period, overall, 561, 542, 452, 479, and 375 students participated in lectures on RO in the 6th to 10th semester, respectively, with median values of 89, 88.5, 76, 78.5, and 63.5 participants for lectures in a single semester (see Table 1 and Fig. 2a for overview). In comparison between the pre-pandemic and pandemic time, there were no significant differences in participation rates (*p* = 0.7, *p* = 0.1, *p* = 0.1, *p* = 0.4, *p* = 1.0 using exact significance in the Mann–Whitney *U*-test for the 6th to 10th semester, respectively). Focusing on students attending > 75% of classes in the respective semester, all semesters revealed an increased rate during the pandemic (median values: 38 vs. 79%; 44 vs. 73%; 45 vs. 64%; 41 vs. 77%; 41 vs. 71%; see Fig. 2b for absolute numbers). This difference was significant between pre-pandemic and pandemic semesters for all semesters analyzed (*p* = 0.046, *p* = 0.05, *p* = 0.05, *p* = 0.05, *p* = 0.05, using asymptotic significance in the Mann–Whitney *U*-test for the 6th to 10th semester, respectively).

**Table 1:** **Participant numbers**. Median absolute participation numbers of the 6th to 10th semester for the pre-pandemic (winter semester 2018/2019 to winter semester 2019/2020) and pandemic period (summer semester 2020 to summer semester 2021). The second column indicates the median number of students attending > 75% of the respective course in each case

	6th		7th		8th		9th		10th	
	Participants	> 75%	Participants	> 75%	Participants	> 75%	Participants	> 75%	Participants	> 75%
Median	89	59.5	88.5	53.5	76	42	78.5	44.5	63.5	34
Median pre-pandemic	87	33	81	33	68	33	78	32	64	27
Median pandemic	105	89	101	67	83	53	90	69	63	45

Median evaluation results were 31.5, 25.25, 22.5, 29.5, and 25.25 on the 101-point Likert scale for each semester (Table 2). Statistical analysis revealed a significant improvement for the 7th and 9th semester in the pandemic semesters in comparison to the pre-pandemic time (22.5 vs. 35; *p* = 0.046 and 25 vs. 32; *p* = 0.05 for the 7th and 9th semesters, respectively). The other semesters showed *p*-values > 0.05 when employing the asymptomatic significance in the Mann–Whitney *U*-test (6th semester: *p* = 0.261; 8th semester: *p* = 0.121; 10th semester: *p* = 0.184). Special focus has been laid on the course “Anatomy and Imaging,” which is obligatory to attend (in contrast to the aforementioned lectures). Therefore, we did not analyze participant numbers but focused on evaluation results, which ranged between 7 (summer semester 2019) and 22 (summer semester 2020) on the 101-point Likert scale, without significant difference between pre- and pandemic semesters (exact significance: *p* = 0.2). The apparent deterioration in evaluation results, albeit not statistically significant, prompted us to extend the period analyzed and to include summer semester 2017, winter semester 2017/2018, and summer semester 2018 in the pre-pandemic period, with median evaluation results of 9, 8, and 11, respectively. There was a significant difference between pre- and pandemic semesters (U = 0.5; Z = −2.112; *p* = 0.035). In general, digital transfer of our lectures received values ranging from −3 to 0 for the clinical semester, with median values of −1.5, 0, 0, 0, and 0 for the 6th to 10th clinical semester, respectively.

**Table 2:** **Evaluation results.** Evaluation results of the 6th to 10th semester according to the semester cohort with standard deviations in parentheses. Evaluation was performed on a 101-point Likert scale (0 the best, 100 the worst grade). There was a significant improvement for the 7th and 9th semester in the pandemic semesters in comparison to the pre-pandemic time (22.5 vs. 35; *p* = 0.046 and 25 vs. 32; *p* = 0.05 for the 7th and 9th semester, respectively)

Semester	6th	7th	8th	9th	10th
WS 2018/2019	32 (23)	35 (21.5)	24 (21.9)	31 (20.9)	33 (21.9)
SS 2019	31 (20.9)	43 (25.6)	24 (18.2)	34 (21.7)	49.5 (24.8)
WS 2019/2020	34.5 (20.7)	28 (21.9)	22 (20.6)	32 (23)	23 (18.5)
SS 2020	26 (18.6)	19.5 (17.2)	16 (15.3)	24 (20.7)	27.5 (18)
WS 2020/2021	32 (20.8)	22.5 (18.1)	17 (17.8)	28 (18.6)	23 (16.5)
SS 2021	31 (20.2)	22.5 (19.8)	23 (19.4)	25 (17.3)	21 (18.1)
Median	31.5	25.25	22.5	29.5	25.25
Median pre-pandemic	32	35	24	32	33
Median pandemic	31	22.5	17	25	23

*SS* summer semester, *WS* winter semester

## Discussion

The hereby presented analysis demonstrates the successful digital transfer of a core curriculum in RO accelerated by the global pandemic. The initial hypothesis of a potential deterioration in participation and evaluation results due to the loss of direct interaction with our students could not be verified. In contrast, there may even be a perspective for structural improvement, with some semesters revealing superior evaluation results. Additionally, there was an increase in the number of students attending > 75% of all lectures.

Participation rate is a key component of teaching as it is a prerequisite for knowledge transfer but also a cardinal component for increasing awareness towards the specialty, e.g., to facilitate recruitment of further residents and doctoral candidates. The increased participation rate in the pandemic period is based on a low threshold for attendance. Students were more motivated to join a (digital) lecture (and follow it completely) in comparison to “traditional” formats. As attendance of lectures is not obligatory, the increased adherence to the courses is likely to reflect true motivation to learn about RO.

At the moment, there are diverging trends concerning participation in our lectures fostered by the hybrid strategy (see “Methods”): while some students return to their alma mater and attend the respective teaching formats in presence, the majority prefer the digital versions and follow the lecture as online streaming. This concept is particularly challenging for the lecturer, who has to pay attention both to the “real” auditorium but also to virtual participants by surveilling the integrated chat function.

Despite its importance, the mere participation rate is not a sufficient parameter for attractive digital education in light of the upcoming *Masterplan Medizinstudium *with its competence orientation. Thus, further analysis focused on advanced learning with integration of interactive or innovative elements to illustrate the evolution of RO teaching.

At our faculty, lectures are enriched with interactive elements such as short polls and multiple-choice questions, which can be answered by an application implemented in the presentation software. Their introduction was rated positively in our free-text evaluation commentary. This may be one reason for the superior evaluation of digitalization in the 6th semester, in which these elements are already established. In the future, amplification of these tools is envisaged. We are currently designing a podcast-based online database providing fundamental knowledge of RO concepts, biology, and physics. The aim of this new tool is to provide students with a structured information source enabling preparation and repetition of the lectures. In addition, the repeated explanation of fundamental concepts of radiation treatment such as intensity-modulated radiation treatment may be avoided, thereby reducing redundancy. Similar screencasts have been developed and established in an e‑learning concept in gynecology and obstetrics and were evaluated positively [16].

To gain an overview of e‑learning at other faculties, the systemic review has been performed. It identified only 11 full-text papers on the subject, demonstrating a paucity of evidence and concepts (Table 3). Some analyses just mention a “virtual conference system for all educational activities” or present possible screen-based simulations without providing details [12, 17].

**Table 3:** Systemic review. Overview of publications on virtual education for medical students in radiation oncology as identified by the systemic review

Author	Concept	Details	Participants	Evaluation
Dapper [18]	Traditional seminars, survey on e‑learning	Five seminars of 45 min	128	Post-course evaluation on participation, acceptance, judgement and effectiveness
Das [12]	Virtual conference system for all educational activities	Not provided	–	Staff’s consent
Franco [19]	Virtual rotation 1 week	Experience of RO, didactic teaching, mentorship opportunities, and capstone experience	12	Evaluation pending
Kahn [20]	Virtual rotation 2 weeks	Contouring cases, structured lectures/didactics	12	Multiple choice testing, pre- and post-clerkship assessment of overall knowledge of RO
Kahn [21]	Virtual panel discussion	Six virtual case-based educational rotations of 1.5–2 h	427	Pre- and post-session evaluation, improvement of RO importance
Kim [22]	Flipped classroom 1 week	Short lectures, interactive tasks (e.g., contouring), (visit of treatment sites)	110	Evaluation of concept and instructors
Phillips [23]	Virtual treatment room	Interactive 3D visualization of patient anatomy, RT planning, and RT delivery in a virtual treatment room	–	Not quantified
Pollom [24] Sandhu [25]	Virtual clerkship 2 weeks	Didactic lectures, virtual clinic, interactive polls, tumor boards, journal club	12 26	Pre- and post-RT rotation, improvement of understanding of RO, evaluation of interest in RO
Rooney [17]	Review of simulator-based learning	Various concepts covering lectures, workshops, only partially web-based concepts	–	Satisfaction, evaluation of importance
Taubert [26]	VR simulation	VR simulation of palliative patient with nausea/vomiting and patient undergoing RT	72	Comfort with and suitability of concept, recommendation, free-text commentaries

*RO* radiation oncology, *RT* radiotherapy, *VR* virtual reality

The remaining papers were further analyzed and may be broadly divided into two main topics, both of which aim at increasing student involvement in teaching but demonstrate different degrees of activation: “virtual away rotations” and “flipped classroom.” The “flipped classroom” is a modern concept in which students are obliged to perform preparatory work before class (e.g., reading a book chapter, listening to a podcast, or watching a video) with the following building upon this knowledge [27, 28]. This creates space for discussions or interactive learning activities [27, 28]. Dapper et al. elaborated a series of traditional seminars but also conducted a survey on the potential introduction of alternative teaching methods and e‑learning [18]. Whereas e‑learning would have been well appreciated, there was a mixed response concerning alternative learning methods (self-study or video clips), with only 42% of students supporting this [18].

Virtual away rotations are designed as full-day online courses of 1–2 weeks in which students visit an RO treatment facility virtually, with participants being introduced to clinical routine and treatments [19, 20, 22, 24, 25]. Typically, they provide tailored didactic lectures or seminars, make participation at clinical conferences, journal clubs, treatment sessions, and consultations possible and may also comprise practical experiences like contouring (see [19, 22, 24] for exemplary timetables). With a modularized curriculum, students may be able to configurate an individualized agenda [22]. Advantages of these concepts lie in cost efficacy, flexibility, and accessibility for the participating students [29]. They offer the possibility to expose large numbers of students to practical RO education and may also aim at addressing underrepresented groups among medical students in order to increase diversity [19, 22, 25]. Although evaluation of some of the concepts is pending, preliminary results reveal a high degree of appreciation and content with this new didactic strategy [20, 22, 25]. An evaluation by Kahn et al. was able to show significant improvement in students’ knowledge of RO from pre- to post-virtual rotation [20]. This included the role of radiation oncologists, physicists, radiation therapists and dosimetrists, training paths, contouring and treatment planning, as well as knowledge on treatment of different entities [20]. These data are supported by an evaluation of Sandhu et al., in which knowledge of and interest in RO could be increased by means of a 2-week virtual clerkship [25]. Still, this does not translate into a rise in the intention to choose RO as a specialty for residency but rather provides basic knowledge [25]. The challenge to attract young medical students to the field of RO and to recruit future doctoral candidates and residents most likely requires a continuous effort and a longitudinal curriculum spanning several semesters.

Apart from the main topics, there are suggestions for short interventions or teaching modules like simulation of a virtual RO treatment room or the experience of a patient undergoing radiation treatment, both provided by 3D virtual reality technology [23, 26].

Kahn et al. validated the application of digital technology in a series of seminars dedicated to different entities and revealed increased understanding after attendance [21]. Again, there was no change in the interest of applying for a RO residency, with a high value both before and after the sessions (78.3 vs. 77.4% for pre- and post-session responses, respectively) [21]. A comparable webinar series has been implemented by the German Society for Radiation Oncology (DEGRO) as an initiative of the Young DEGRO working group and presents monthly sessions on varying cancer sites as well as topics of radiation biology and physics.

Regarding curricular development in RO, the DEGRO has defined a core schedule covering all relevant topics till board certification [30]. However, the ongoing political measures aiming at competence-based education demand the definition and adaption of a modern curriculum [31]. A recent consensus paper of the consortium academic radiation oncology of DEGRO introduces digital or hybrid seminars as a putative teaching format to address interdisciplinary oncology lectures or seminars on different entities, whereas a flipped classroom model may be used for case discussions [31].

Overall, the available literature illustrates feasible and efficient digital projects, which reveal a high degree of innovation, creativity, and competence orientation. The heterogeneity of concepts likely mirrors the variability of didactic situations, with no possible one-fits-all approach to e‑learning. Pivotal questions for the implementation of digital formats are the following: “Which extent of digitalization should be pursued (total vs. partial curriculum)?”; “Which courses are to be transferred (lectures vs. seminars/practical training)?”; and “What degree of students’ active learning is requested (lectures vs. flipped classroom with obligatory preparation)?”

Therefore, digital concepts have to be tailored to the individual situation, but may prove to be two-sided swords: in view of the global pandemic, they enable or even deepen university teaching. In contrast, they deprive students of direct interaction with real patients, which induces the fear of lacking practical experience [32]. Consequently, a careful balance between virtual and real-world education has to be maintained, combining the best of both strategies. This is corroborated by a meta-analysis in which combined strategies of e‑learning and traditional teaching in medical education turn out to be superior to the established practices of teaching [33].

Being a retrospective and monocentric evaluation, our analysis does not shed light on other university hospitals in Germany. This is of particular importance as education is legislated at the federal state level in Germany, which is known to cause heterogeneity. Concerning the systemic review, a substantial publication bias has to be suspected, with only studies with positive results being published. Furthermore, data on efficacy of the respective concepts are infrequently given, demanding a further structured evaluation in the future. This is also true for our own mono-institutional evaluation, as we did not analyze consecutive examination results after digital education.

It remains uncertain how much of the digital progress initiated by the COVID-19 pandemic will prevail. Anyhow, the global crisis has taught us the value of digital tools and education. It will be our task to integrate these new technologies within established concepts and to evolve medical education in the future.
